# Development and Clinical Validation of an Artificial Intelligence-Based Automated Visual Acuity Testing System

**DOI:** 10.3390/life16020357

**Published:** 2026-02-20

**Authors:** Kelvin Zhenghao Li, Hnin Hnin Oo, Kenneth Chee Wei Liang, Najah Ismail, Jasmine Ling Ling Chua, Jackson Jie Sheng Chng, Yang Wu, Daryl Wei Ren Wong, Sumaya Rani Khan, Boon Peng Yap, Rong Tong, Choon Meng Kiew, Yufei Huang, Chun Hau Chua, Alva Khai Shin Lim, Xiuyi Fan

**Affiliations:** 1 Department of Ophthalmology, National Healthcare Group Eye Institute, Tan Tock Seng Hospital, Singapore 308433, Singapore; 2 Center of AI in Medicine, Lee Kong Chian School of Medicine, Nanyang Technological University, Singapore 308232, Singapore; 3 College of Computing and Data Science, Nanyang Technological University, Singapore 639798, Singapore; 4 School of Electrical and Electronic Engineering, Nanyang Technological University, Singapore 639798, Singapore; 5 Singapore Institute of Technology, Singapore 828608, Singapore; 6 Digital and Smart Health Office, National Healthcare Group, Singapore 308443, Singapore

**Keywords:** automated, artificial intelligence, visual acuity test

## Abstract

Background: To develop and validate an automated visual acuity (VA) testing system integrating artificial intelligence (AI)–driven speech and image recognition technologies, enabling self-administered, clinic-based VA assessment; Methods: The system incorporated a fine-tuned Whisper speech-recognition model with Silero voice activity detection and pose estimation through facial landmark and ArUco marker detection. A state-driven interface guided users through sequential testing with and without a pinhole. Speech recognition was enhanced using a local Singaporean English dataset. Laboratory validation assessed speech and pose recognition performance, while clinical validation compared automated and manual VA testing at a tertiary eye clinic; Results: The fine-tuned model reduced word error rates from 17.83% to 9.81% for letters and 2.76% to 1.97% for numbers. Pose detection accurately identified valid occluder states. Among 72 participants (144 eyes), automated unaided VA showed good agreement with manual VA (ICC = 0.77, 95% CI 0.62–0.85), while pinhole VA demonstrated moderate agreement (ICC = 0.63, 95% CI 0.25–0.83). Automated testing took longer (132.1 ± 47.5 s vs. 97.1 ± 47.8 s; *p* < 0.001), but user experience remained positive (mean Likert scale score 4.3 ± 0.8); Conclusions: The AI-based automated VA system delivered accurate, reliable, and user-friendly performance, supporting its feasibility for clinical implementation.

## 1. Introduction

Vision is one of the most important human senses. Daily interaction with the environment depends heavily on good vision, and vision loss can profoundly affect function and quality of life [1]. Evaluation of visual function is therefore a key component of the ophthalmic examination, providing invaluable information for diagnosis, investigation, and management. Early and accurate identification of visual impairment is critical for maintaining optimal ocular health.

Visual acuity (VA) is a fundamental measure of visual function and an integral part of routine eye examinations. It quantifies an individual’s ability to distinguish symbols or letters at a standardized distance, typically 4 to 6 m. Several methods are commonly used to assess VA, including the Snellen chart, tumbling E, and Landolt C [2]. These charts evaluate an individual’s capacity to resolve two spatially separated stimuli, thereby determining the level of VA. Monitoring VA is central to clinical management and frequently serves as a vital outcome measure in clinical research [3].

Traditionally, VA testing is conducted by an optometrist or trained healthcare worker, who is responsible for: (1) instructing and ensuring that the patient covers the correct eye or uses a pinhole occluder if necessary, (2) switching between optotype sizes, and (3) calculating and recording the VA scores [4]. However, this process is labor-intensive, time-consuming, and dependent on operator consistency. Variability in staff training and expertise may also affect VA measurement accuracy [5].

In recent years, driven in part by the COVID-19 pandemic, novel methods of measuring VA have emerged, including mobile applications and virtual or augmented reality technologies [6,7]. The benefits of these platforms include increased accessibility, ease of use, and reduced manpower needs, potentially lowering healthcare costs [8,9].

Nevertheless, several limitations remain. Mobile devices may produce glare that affects VA results [10], and their limited screen sizes restrict the range of optotype sizes available for testing [11]. Augmented reality systems may lack sufficient effective resolution to accurately assess patients with good vision [12]. Additionally, both virtual and augmented reality devices often rely on multiple-choice letter selection, allowing for guessing rather than true recognition [12,13].

This study is split into four phases: (1) technical development of the visual acuity system, (2) speech recognition development, (3) pose recognition validation, and (4) clinical validation. It aims to automate VA testing by integrating pose monitoring and speech-recognition technologies into a self-administered platform. Through the integration of various artificial intelligence algorithms into a single platform, we seek to develop and validate an artificial intelligence-based system that enables users to independently perform accurate VA assessments without external user intervention, thereby enhancing clinical workflow efficiency and accessibility of vision testing.

## 2. Materials and Methods

We will discuss the development of the visual acuity system, which is namely split into four phases: (1) the technical development of the visual acuity system, (2) speech recognition validation, (3) pose recognition validation, and (4) clinical validation.

### 2.1. Technical Development of the Visual Acuity System

#### 2.1.1. Design of Automated Visual Acuity Measurement

The automated VA measurement system was modeled after the standard VA measurement protocol. Participants were seated 4 to 6 m from the VA chart display [14]. With one eye occluded at a time, participants read optotypes displayed on the screen. In clinical settings, VA is typically tested from the largest optotype (Snellen chart 6/120) to the smallest standardized line (Snellen chart 6/6) [15].

To improve testing efficiency, only the first character of each row was shown until the participant was unable to identify it. The entire row of five optotypes from the last correctly identified single optotype was then presented. Participants were required to correctly identify more than half the optotypes in a row to progress to the next smaller line [14,16]. If the participant failed to read a line above 6/9, a pinhole occluder was introduced. The VA was documented as the smallest line that could be correctly read. Additional or missing optotypes were recorded as +1, +2, −1, or −2 adjustments [17].

#### 2.1.2. Speech Inference Development

The system employed the pre-trained Whisper model [18] for automatic speech recognition (ASR). Whisper is a multi-task encoder–decoder-based speech model trained on multilingual, weakly labeled web data. To optimize response time, the small model variant was used. To achieve real-time speech recognition, voice activity detection (VAD) was implemented using the Silero VAD model [19]. It identifies speech segments within the audio stream, feeding only speech portions to Whisper for recognition. While automatic speech recognition systems work well in a general controlled environment, practical applications often face challenges due to the variability introduced by different speakers and noisy recording environments.

To enhance the robustness of ASR accuracy under real-world conditions, we employed vocabulary constraints and accent adaptation. In the Snellen chart, only nine English letters or digits 0 to 9 are used. For our VA system, vocabulary was restricted to the English alphabet (A–Z), digits (0–9), and limited control commands (“OK”, “Blurred”, “Skip”) to minimize recognition errors. To adapt to Singaporean English accents, the Whisper model was fine-tuned using 2000 locally collected speech samples of alphabets and numbers, employing the Low-Rank Adaptation (LoRA) technique [20].

#### 2.1.3. Face Pose Detection and Validation Engine

The pose-detection system integrated several computer vision techniques for real-time face pose estimation relative to the occluder device. Video frames from a webcam were undistorted using camera calibration data and then zoomed, cropped, and centered on the user’s face. The preprocessed frame was analyzed in parallel by:

Facial Landmark Detection: A pre-trained MediaPipe model asynchronously identified key facial features (eyes, nose, mouth, face contour) and computed metrics such as iris centers and face dimensions for pose analysis and adaptive camera control.

**Figure 1:**
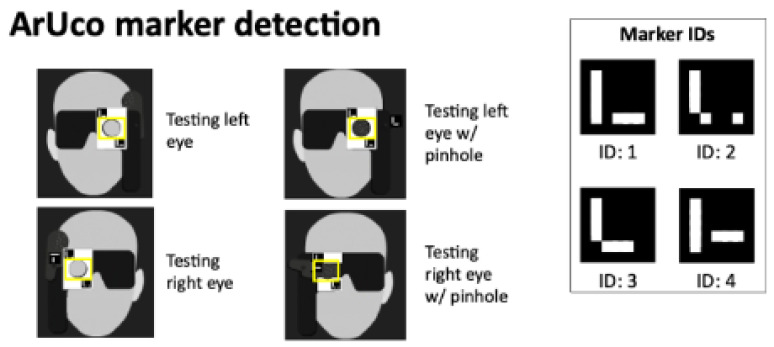
ArUco markers affixed to the occluder identify four states—left eye, left eye with pinhole, right eye, and right eye with pinhole—enabling real-time validation of user posture and occluder position.

**Figure 2:**
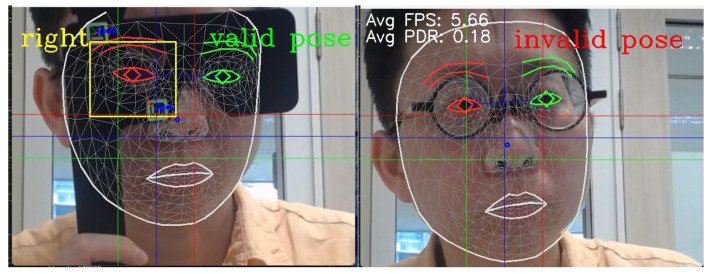
Examples demonstrating valid and invalid participant poses based on real time evaluation of occluder position. Facial landmark detection was performed using a pre-trained MediaPipe model.

ArUco Marker Recognition: The frame was processed using adaptive thresholding and contour detection, with contrast enhancement via histogram equalization or CLAHE, to highlight potential marker regions. The OpenCV ArUco library detected four marker configurations on the 3D-printed occluder: (i) left eye; (ii) left eye with pinhole; (iii) right eye; and (iv) right eye with pinhole (Figure 1). This allowed the system to infer occluder state and compute a bounding box to verify that the corresponding iris falls within the correct region and validate that the correct eye was covered (Figure 2).

**Figure 3:**
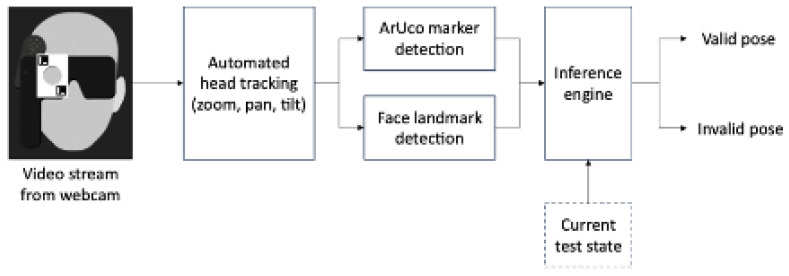
Overview of the face pose detection workflow, detailing the sequential steps used to extract facial landmarks, analyze occluder position, and determine pose validity.

Additionally, camera pan, tilt, and zoom adjustments were dynamically controlled based on face contour metrics, smoothed with moving averages. The system visualized facial landmarks, marker outlines, and bounding boxes in real time, transmitting pose and occluder status to the server via HTTP for every captured frame (Figure 3).

#### 2.1.4. State-Driven, Adaptive Approach to Comprehensive Eye Examination

A state machine architecture guided the self-administered examination. The process began with the user scanning the identity card (IC) and receiving instructions on correct posture and occlusion. The system then presented progressively smaller optotypes for each eye, starting with the right eye, when the correct response was received. Real-time audio processing via the Whisper ASR model transcribed verbal responses, and the StateManager class dynamically adjusted test progression, including initiation of pinhole testing when indicated.

A flask-based interface displayed optotypes and interactive prompts, updating in real time to maintain a seamless user experience. Upon completion, the system generated a summary report including VA scores, a printable barcode, and optional user feedback. The multi-threaded, event-driven design allowed simultaneous handling of user input, audio processing, and state transitions.

#### 2.1.5. User Interface Development

The graphical user interface (UI) for both the main display showing optotypes and the handheld device was developed using HTML and JavaScript modules dynamically rendered using the Jinja Template Engine. The UI closely mirrored the manual testing workflow.

Users initiated the test through a touch interface and received voice-guided instructions to position the occluder correctly. The real-time pose monitoring system verified alignment, posture, and occlusion. The user could select either a number-based or letter-based VA test. Identity verification was achieved by scanning the IC before the test began. Instructions and animations were displayed on both the main monitor and handheld device to guide posture and correction. Test initiation required verbal confirmation (“OK”).

Several features were implemented to account for potential human errors. A “repeat” command was implemented to allow re-testing if a speech recognition error occurred. If the user required assistance, an alert interface allowed staff intervention without terminating the session.

The detailed flowchart of the self-administered VA test process is provided in the Supplementary Materials.

### 2.2. Speech Recognition Validation

ASR performance was evaluated using speech samples collected from 11 speakers in a simulated VA test environment. Each participant read 30 randomly generated alphanumeric sequences using two microphones under both quiet and noisy conditions, yielding 120 utterances per participant.

A comparative analysis was performed between the original Whisper model and the fine-tuned model. Word error rate (WER) was employed as the evaluation metric. It measured the number of errors (substitutions, insertions, and deletions) made by the ASR system compared to the ground truth. WER was calculated as follows:

WER = 100 × (*S* + *I* + *D*)/*N*

where *S* = substitutions, *I* = insertions, *D* = deletions, and *N* = total words in the ground truth transcription. A lower WER value indicates better ASR performance.

### 2.3. Pose Recognition Validation

Pose detection accuracy was assessed using nine predefined poses: four valid and five invalid (Table 1). The system’s reliability was determined by comparing ArUco marker detection results with expected occluder states.

### 2.4. Clinical Validation Methodology

Prototype validation was conducted at the Tan Tock Seng Hospital Eye Clinic. Participants were recruited from the standard VA testing queue. Inclusion criteria were: (1) manual VA better than counting fingers, and (2) ability to understand and speak English to ensure they had visual and vocal function for accurate comparison.

Performance metrics included:

- Accuracy: Agreement between prototype and manual VA results.
- Efficiency: Time required for each test.
- User Satisfaction: Participant feedback on usability and satisfaction.

### 2.5. Statistical Analysis

Data analysis for the clinical validation was performed using R statistical software (Version 4.3.0). Data were described as mean (standard deviation) and median (interquartile range). Snellen VA was converted to logMAR using the formula based on the paper by Tiew et al. [21]. VA data were assumed to be independent between eyes. The test of normality was conducted using the Shapiro–Wilk test. Agreement between manual and automated VA was estimated using Bland–Altman plots to assess biases with 95% limits of agreement. The intraclass correlation coefficient (ICC) was also computed to measure inter-method agreement. A Wilcoxon signed-rank test evaluated any significant differences in VA and testing time between methods, with the *p*-value set at 0.05. Correlations were analyzed using Pearson’s if the results were normally distributed, or Spearman’s if the results were not normally distributed. Repeated measures ANCOVA was used to determine whether covariates influenced testing duration.

### 2.6. Ethics Approval

The study was reviewed and approved by the National Healthcare Group Domain Specific Review Board (DSRB) (approval number: 2024/00157, date of approval: 25 April 2024). The research was conducted in accordance with the ethical standards outlined in the Declaration of Helsinki and local ethical regulations. Written informed consent was obtained from all participants. Data confidentiality and participant privacy were strictly maintained, with additional safeguards applied for vulnerable populations.

## 3. Results

The results were analyzed according to the phase of the study, with evaluations of speech recognition, pose detection, and clinical validation with real-world participants.

### 3.1. Laboratory Validation of Speech Recognition

The fine-tuned Whisper model achieved substantially improved recognition accuracy compared with the baseline model. The WER for letters decreased from 17.83% to 9.81%, and for numbers, from 2.76% to 1.97%, demonstrating the effectiveness of fine-tuning for the VA test vocabulary.

### 3.2. Laboratory Validation of Pose Detection

Pose determination was based on the detection of ArUco marker IDs. Results in Table 2 showed that valid poses were consistently recognized, whereas invalid poses produced either incorrect or null outputs, confirming the system’s ability to accurately verify left-, right-, and pinhole-based occluder poses.

### 3.3. Clinical Validation

**Figure 4:**
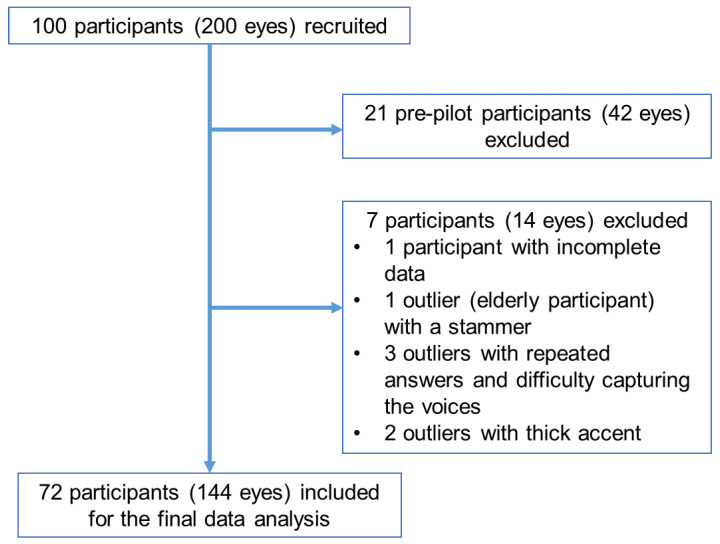
Selection of eyes included in the data analysis.

A total of 100 participants were recruited. After excluding 21 pre-pilot participants (42 eyes) enrolled before the optimized speech-recognition system and one participant (2 eyes) with incomplete data, 72 participants (144 eyes) were included in the analysis (Figure 4).

Among these, we identified 10 “outliers”, defined as participants with a logMAR VA difference of ≥0.4 between manual and automated VA in either eye. Six outliers (12 eyes) were excluded for valid clinical reasons: one elderly participant with a stammer, three with repeated answers and difficulty capturing the voices, and two with thick accents that impaired voice capture.

The final dataset comprised 72 participants (35 females (48.6%) and 37 males (51.4%)) with a mean age of 60.0 ± 12.2 years (range, 26.0–81.0 years).

The mean manual VA test duration was 97.1 ± 47.8 s, compared with 132.1 ± 47.5 s for the automated system (*p* < 0.001). The mean accuracy of the automated VA system was 81.8 ± 13.2%. Average user experience feedback was 4.3 ± 0.8 on a 5-point Likert scale.

**Figure 5:**
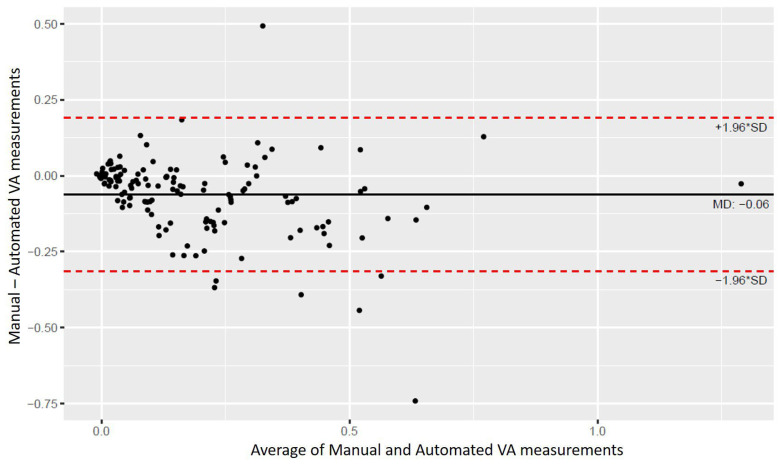
Bland–Altman analysis comparing manual and automated VA measurements (without pinhole).

Bland–Altman analysis demonstrated good agreement between manual and automated VA measurements. For VA without pinhole (Figure 5), the mean difference was −0.06 ± 0.13 logMAR, with 95% limits of agreement from −0.31 to 0.19 logMAR, and an intraclass correlation coefficient (ICC) of 0.77 (95% CI, 0.62–0.85), indicating strong concordance. The automated system tended to record slightly better VA scores compared with manual testing (median 0.14 vs. 0.09 logMAR; *p* = 0.016)

**Figure 6:**
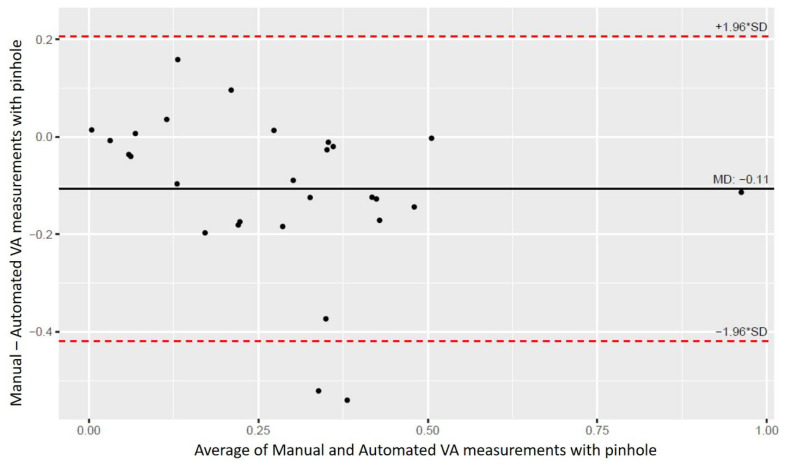
Bland–Altman analysis comparing manual and automated VA measurements (with pinhole).

For VA with pinhole (Figure 6), the mean difference was −0.11 ± 0.16 logMAR, with limits of agreement from −0.42 to 0.21 logMAR, and a moderate ICC of 0.63 (95% CI, 0.25–0.83). This indicates reasonable agreement, albeit with wider variability, likely due to the smaller sample size (28 eyes).

Subgroup analysis showed no correlation between participant age and the time difference between manual and automated testing (Spearman’s ρ = 0.06, *p* = 0.963). Repeated-measures ANCOVA with age as a covariate revealed F = 0.882, *p* = 0.639, confirming that age was not a significant factor influencing test duration.

## 4. Discussion

VA assessment remains a cornerstone of ophthalmic examination, essential for monitoring disease progression and guiding management [22]. However, the time and manpower required for conventional VA testing continue to constrain clinic efficiency. Artificial intelligence offers opportunities to streamline clinical workflows and enhance care delivery [23]. This study presents the development and validation of an automated VA assessment station integrating computer vision and speech recognition technologies to enable self-administered, accurate testing.

### 4.1. Performance of the Automated VA System

Our findings suggest that our automated VA system demonstrated performance comparable to that of manual VA testing. Overall, the Bland–Altman plots show that most data points lie within the 95% limits of agreement for both VA without and with a pinhole. The small negative mean differences suggest that the automated VA system slightly overestimated VA compared with manual testing, but within clinically acceptable limits. Moderate agreement for VA with pinhole was likely secondary to the smaller sample size and increased test complexity in that subgroup.

The automated VA test required a significantly longer completion time than the manual method. This delay is likely attributable to participants’ unfamiliarity with the novel interface and workflow. In Singapore, manual VA testing is routinely encountered through community and occupational screening programs [24,25] and is typically conducted by allied health professionals, whereas the automated system introduces new voice-guided instructions and self-operated occlusion steps that are new to participants. A brief familiarization or practice session could reduce this learning effect in future iterations. Increased familiarity with the system through repeated encounters or clinic visits, along with its wider adoption in clinical settings, may also shorten testing duration over time.

Despite the longer testing time, user satisfaction was high, with an average rating of 4.3 ± 0.8 on a five-point Likert scale. This positive reception suggests that the system’s usability and clarity of guidance compensated for its novelty and longer testing duration, which is an encouraging indicator for long-term clinical adoption.

### 4.2. Optimization of Speech Recognition

Accurate verbal recognition is critical for self-administered VA testing. Speech variability among Singaporean English speakers, including accent diversity, articulation speed, intonation, and pronunciation of words, posed challenges for ASR, potentially impacting the accuracy and speed of the test conducted [26]. Models trained predominantly on Western English corpora often generalize poorly to this local linguistic context.

Fine-tuning of the Whisper model using a constrained vocabulary (A–Z, 0–9, and limited control commands) and locally collected speech samples substantially improved recognition accuracy, reducing the word-error rate by 8.02% for letters and 0.76% for numbers. Nonetheless, residual inaccuracies persisted among participants with strong accents or disfluency, leading to the exclusion of six outliers. We aim to further train the model on a larger local data set to further enhance the robustness of the speech recognition model.

Ambient noise present in the clinical setting was also observed to interfere with voice capture and transcription. Incorporating Silero VAD before Whisper processing effectively filtered non-speech segments and improved transcription reliability. The combined ASR architecture provided a functional solution capable of real-time response within the clinic environment.

### 4.3. Integration of Computer Vision Techniques

Our automated VA system leveraged multiple computer vision methods—facial landmark detection, ArUco marker recognition, and adaptive image processing—to ensure accurate pose validation and occluder positioning. Real-time tracking with automated pan-tilt-zoom adjustments enhanced user alignment and improved system reliability.

To our knowledge, this is the first self-administered VA testing system to incorporate pinhole assessment. Despite the small sample size of pinhole testing in our study (n = 28), results demonstrated moderate agreement between manual and automated measurements (ICC = 0.63; 95% CI, 0.25–0.83), supporting the feasibility of integrating pinhole testing to expand the clinical utility of the automated VA system. The use of a pinhole in VA testing is clinically valuable, as it helps identify refractive errors as potential confounders and can reduce the false-positive rate of VA screening failures by more than half [27].

Limitations of computer vision techniques include potential marker detection errors under extreme illumination, such as dim light or strong glare, or partial occlusion. Although adaptive thresholding, contrast enhancement, and preprocessing techniques in the code mitigated most issues, maintaining clean and undamaged markers on the 3D-printed occluder remains essential. We hope to explore other measures in the future to improve the robustness of the system.

### 4.4. Comparison with Existing Automated VA Models

Previous automated VA systems have primarily focused on home-based self-testing or teleophthalmology applications, or have been developed for pediatric patients [28,29,30,31]. Other digital or semi-automated approaches, such as optotype projection systems and mobile-based VA assessment tools, have demonstrated the feasibility of using a speech-recognition system but still rely on user-driven input. They also lack integrated pose and speech monitoring [32,33]. One study utilized a head-mounted display, showing one optotype at a time, purportedly with the ability for self-administration. It, however, lacked the ability for pinhole testing and demonstrated low accuracy [12]. Another study utilized speech-based visual acuity assessment with a good accuracy of 91.88%. Similarly, there was no pose monitoring, nor the ability for pinhole assessments [34]. Without recognizing, via pose estimation, if the patient was conducting the test properly, or the ability to test pinhole vision, the conduct of the visual acuity test may give inaccurate results. Our study demonstrates an artificial intelligence-driven, clinic-integrated VA station designed for adults in clinical settings, which does not require external user interventions. The combination of speech and vision recognition enables a higher level of automation, including occluder positioning and pinhole assessment, which is rarely incorporated in self-administered models. This dual-modal approach represents a novel step toward fully autonomous visual function testing, and our findings showed encouraging performance supporting its potential for wider clinical adoption.

### 4.5. Challenges in Patient Guidance and Occluder Handling

Although the testing sequence closely mirrored the manual process, some participants, especially elderly and technologically unfamiliar users, experienced difficulty positioning the occluder correctly, resulting in failure to complete the assessment. Incomplete or improper coverage of one eye may compromise accuracy.

Enhancements such as proper user education, animated visual cues, step-by-step audio guidance, and real-time feedback prompts could improve compliance and minimize user error.

### 4.6. Technical and Resource Limitations

Achieving low-latency inference for both speech and pose recognition remains computationally demanding. Systems lacking GPU acceleration may experience lag, affecting test smoothness. Future optimization through model quantization, GPU scheduling, or edge computing (on-device or cloud) deployment could enhance performance and scalability, enabling wider clinical implementation.

### 4.7. Future Directions

Future validation should involve deployment across multiple automated VA stations in real-world clinical environments to assess throughput, integration, and user acceptance among both patients and staff. Longitudinal evaluation may clarify learning effects and workflow adaptation over repeated use. Expanding training datasets for both speech and pose recognition models will further improve accuracy and generalizability. Integration with electronic medical record systems and the addition of remote testing capabilities could further extend clinical utility. In Singapore’s multilingual context, future model development should also incorporate recognition of commonly spoken languages beyond English to ensure practicality and inclusivity in routine clinical use.

## 5. Conclusions

This study presents a novel automated VA testing system that leverages deep learning-based speech and image recognition technologies. The system demonstrates the feasibility of integrating a multimodal artificial intelligence approach into an automated VA test, incorporating speech recognition and pose estimation capabilities. Some limitations exist with the system, including difficulty in conducting the test for elderly and technologically unfamiliar users. Further test–retest evaluations should be considered to determine if familiarity improves usability and reduces the time taken for the evaluation. In this pilot validation, we achieved good agreement with manual testing and satisfactory testing times and user experience, supporting its potential for real-world clinical implementation and broader adoption in ophthalmic practice.

## Figures and Tables

**Table 1:** Definition of valid and invalid poses used for pose-detection validation.

ID	Validity	Pose	Description
1	Valid	Left	Occluder covering right eye (testing left)
2	Valid	Right	Occluder covering right eye (testing right)
3	Valid	Left Pinhole	Left eye with pinhole
4	Valid	Right Pinhole	Right eye with pinhole
5	Invalid	Partial Eye Cover (L)	Occluder not fully covering left eye
6	Invalid	Partial Eye Cover (R)	Occluder not fully covering right eye
7	Invalid	Pinhole Partially Lowered (L)	Incomplete lowering of pinhole on left
8	Invalid	Pinhole Partially Lowered (R)	Incomplete lowering of pinhole on right
9	Invalid	No Occluder	Occluder not raised

**Table 2:** Results of pose detection showing correspondence between detected ArUco marker IDs and final pose classification.

ID	Validity	Pose	Aruco Marker IDs Detected	Final Pose
1	Valid	Left	1	Left
2	Valid	Right	2	Right
3	Valid	Left Pinhole	1, 3	Left_ph
4	Valid	Right Pinhole	2, 4	Right_ph
5	Invalid	Partial Eye Cover (L)	1	Invalid
6	Invalid	Partial Eye Cover (R)	2	Invalid
7	Invalid	Pinhole Partially Lowered (L)	1	Left
8	Invalid	Pinhole Partially Lowered (R)	4	Right
9	Invalid	No Occluder	None	Invalid

## Data Availability

The data presented in this study are available on request from the corresponding author due to privacy or ethical restrictions.

## Supplementary Materials

The following supporting information can be downloaded at: https://www.mdpi.com/article/10.3390/life16020357/s1, Figure S1: Detailed flowchart of the self-administered VA test process.
