# Supplementary figures and images for: Development and Clinical Validation of an Artificial Intelligence-Based Automated Visual Acuity Testing System
Source: Life (Basel). 2026 Feb 20;16(2):357. doi: 10.3390/life16020357 (PMC12942385; doi:10.3390/life16020357)

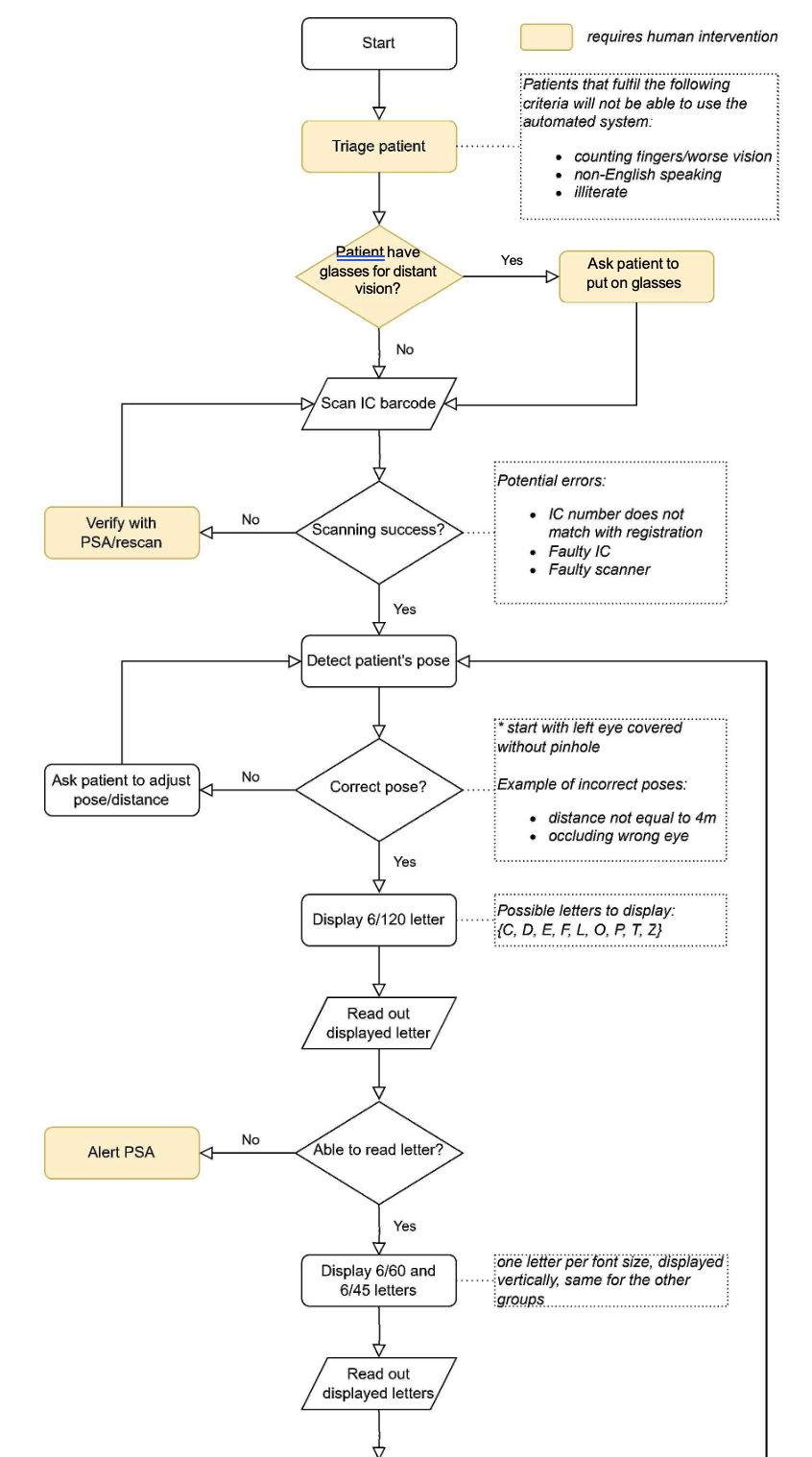

Supplement: Supplementary file 1 [file life-16-00357-s001.zip › Figure 1_part 1.png]

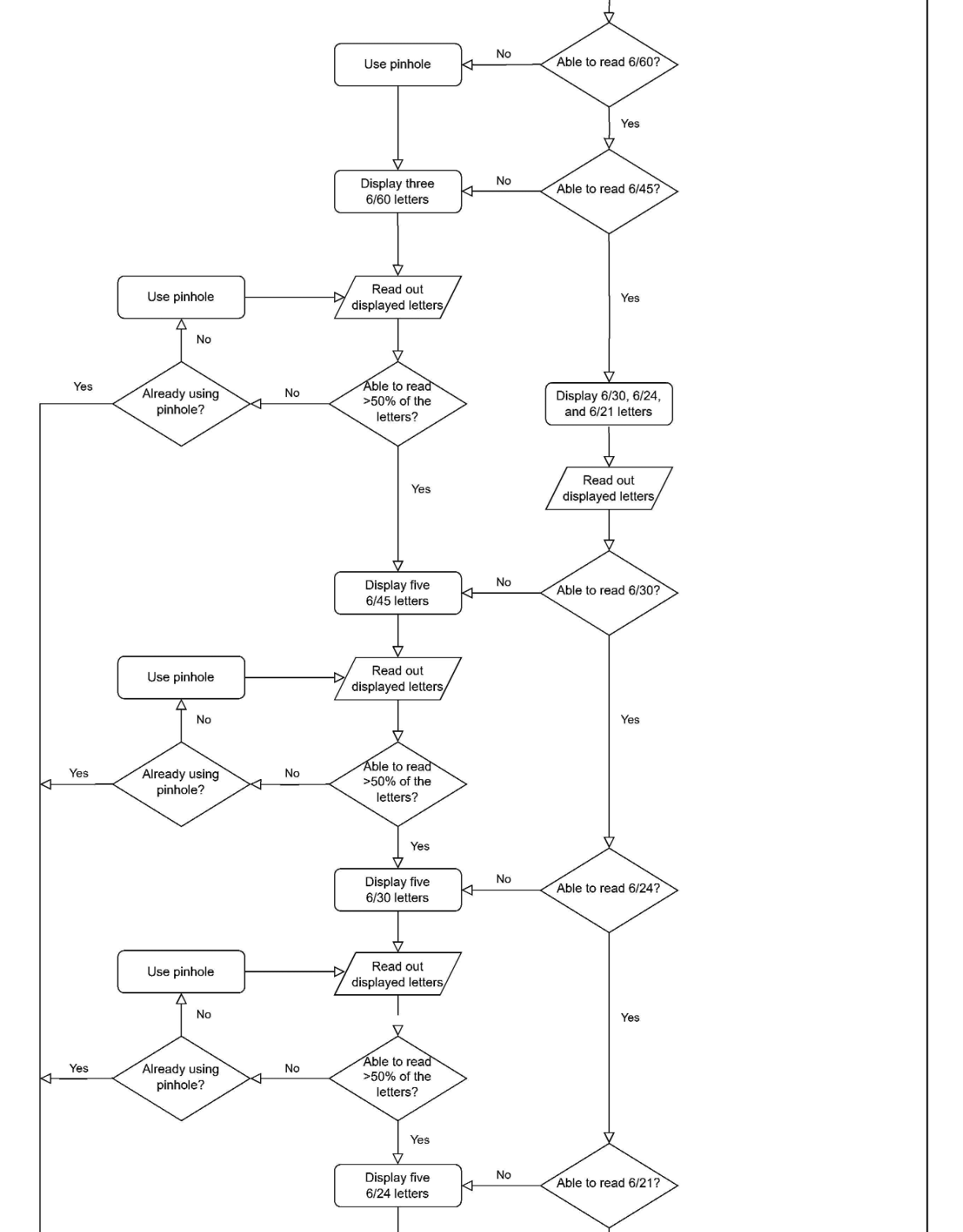

Supplement: Supplementary file 1 [file life-16-00357-s001.zip › Figure 1_part 2.png]

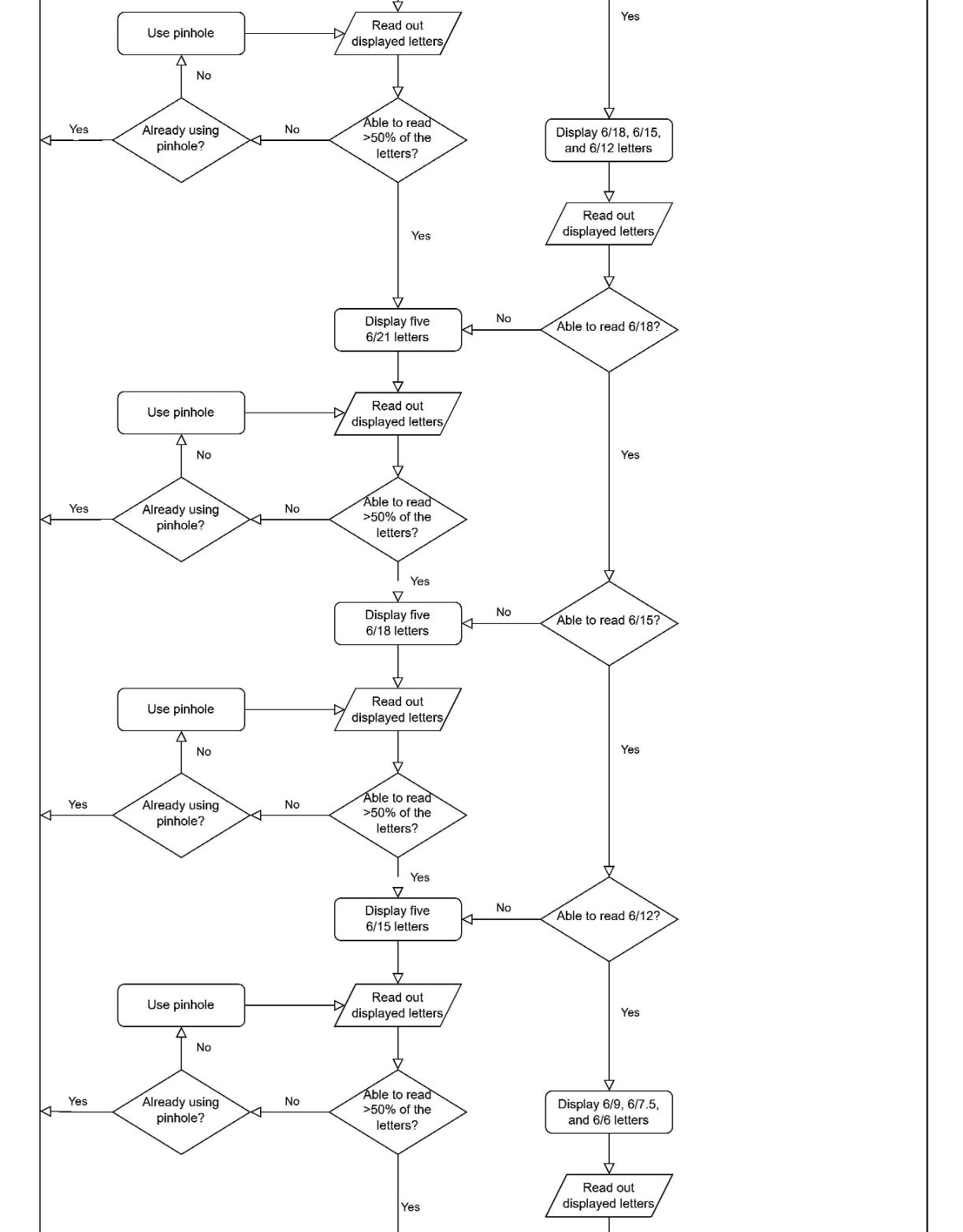

Supplement: Supplementary file 1 [file life-16-00357-s001.zip › Figure 1_part 3.png]

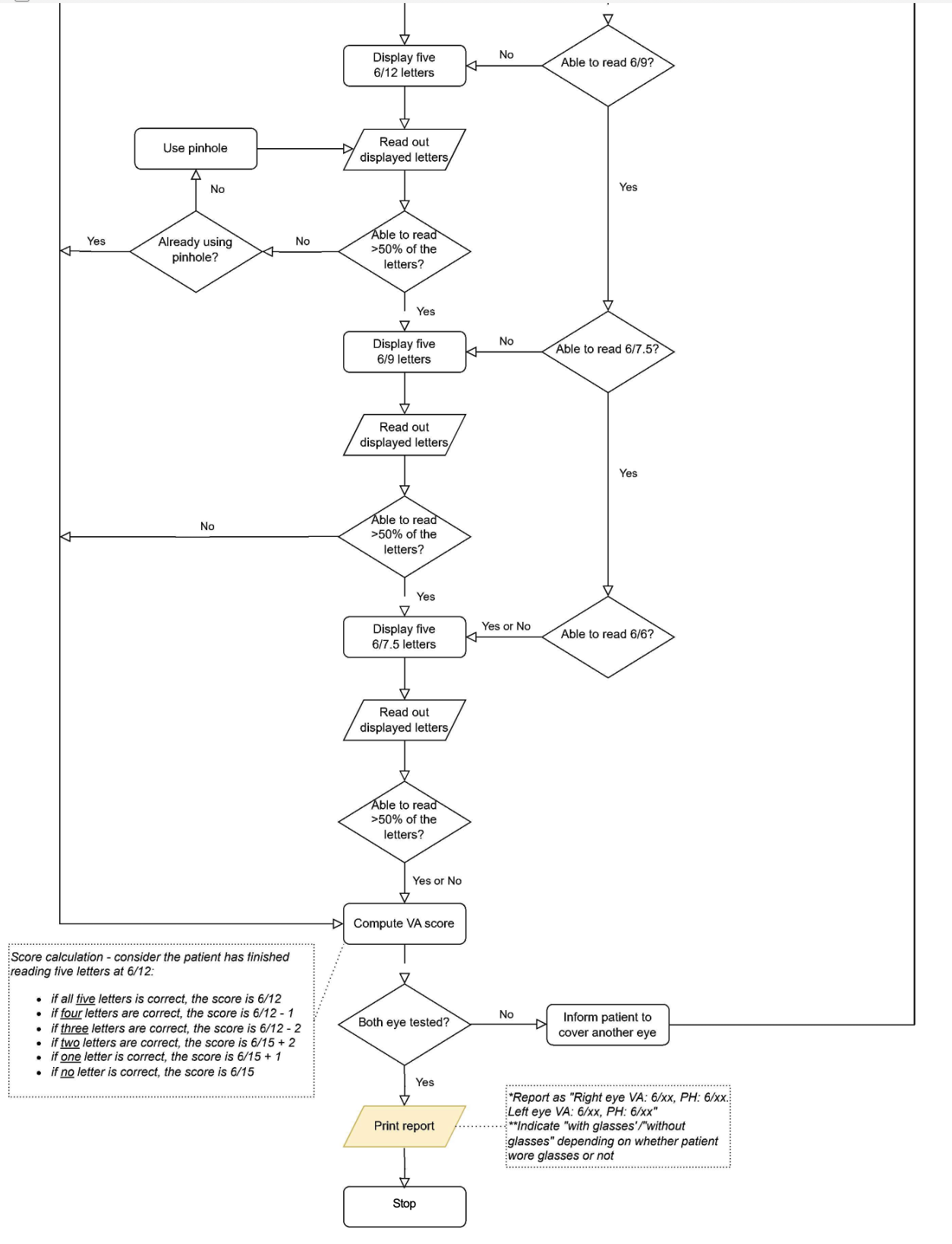

Supplement: Supplementary file 1 [file life-16-00357-s001.zip › Figure 1_part 4.png]
